# The role of adipokines in systemic sclerosis: a missing link?

**DOI:** 10.1007/s00403-019-01893-1

**Published:** 2019-02-26

**Authors:** Jakub Żółkiewicz, Anna Stochmal, Lidia Rudnicka

**Affiliations:** Department of Dermatology, Medical University of Warsaw, Koszykowa 82A, 02-008 Warszawa, Poland

**Keywords:** Adipokines, Adiponectin, Resistin, Leptin, Visfatin, Chemerin, Pathogenesis, Systemic sclerosis, Vaspin, Adipsin, Apelin, Omentin, CTRP-3

## Abstract

Systemic sclerosis is a multiorgan autoimmune disease characterized by vasculopathy and tissue fibrosis of unknown etiology. Recently, adipokines (cell signaling proteins secreted by adipose tissue) have attracted much attention as a cytokine family contributing to the various pathological processes of systemic sclerosis. Adipokines, such as leptin, adiponectin, resistin, adipsin, visfatin or chemerin are a heterogenic group of molecules. Adiponectin exhibits anti-fibrotic features and affects inflammatory reactions. Leptin promotes fibrosis and inflammation. Resistin was linked to vascular involvement in systemic sclerosis. Visfatin was associated with regression of skin lesions in late-stage systemic sclerosis. Chemerin appears as a marker of increased risk of impaired renal function and development of skin sclerosis in the early stage of systemic sclerosis. Vaspin was indicated to have a protective role in digital ulcers development. Novel adipokines—adipsin, apelin, omentin and CTRP-3—are emerging as molecules potentially involved in SSc pathogenesis. Serum adipokine levels may be used as predictive and diagnostic factors in systemic sclerosis. However, further investigations are required to establish firm correlations between distinct adipokines and systemic sclerosis.

## Introduction

Systemic sclerosis (SSc) is an autoimmune connective tissue disease. It is characterized by a chronic course, significantly affecting length and quality of life [55, 56]. The hallmarks of SSc are progressive skin thickening and visceral fibrosis associated with atrophy of subcutaneous tissue, vascular involvement as well as immune dysregulation [58]. The pathogenesis of SSc is still not clearly understood. Genetic, vascular, autoimmune and environmental factors are postulated to have an impact on SSc development [115].

Adipose tissue is believed to be one of the largest endocrine organs in humans [53]. Adipocytes are metabolically active cells and their products are called adipokines. Adipokines are a non-homogenous group of proteins, which can be subdivided, according to their mechanism of action, into auto-, para- and endocrine hormones [52]. The group of adipokines includes: adiponectin, resistin, leptin, visfatin, chemerin, vaspin and many more, including cytokines (IL-6, TNF-α), coagulation factors (PAI-1), growth factors (VEGF, TGF-β) or complement system proteins (adipsin) [4]. Adipokines play a vital role in homeostasis and every disharmony in this precise system may contribute to the development of various diseases such as hypertension or type 2 diabetes [30]. However, the link between adipokines and SSc is still discussed. The aim of this review is to analyze and summarize current data related to the role of adipokines in the pathogenesis of SSc, future perspectives and potential directions for investigations.

## Adipose tissue in systemic sclerosis

Adipose tissue seems to play a crucial role in skin homeostasis and remodeling [101]. Furthermore, degradation of intradermal adipose tissue precedes the onset of dermal fibrosis [74]. Positive feedback loop is suspected wherein adipose tissue is a source of factors exacerbating fibrosis and its replacement by fibroblasts enhances collagen fibers production.

### Adipose tissue and immune system

Adipose tissue stays in a close relationship with the immune system. Adipokines are considered to modulate immune response and interdependence between both systems has been reported to date [34]. Adipokines affect activation and attraction of many immune cells which results in accumulation and differentiation of CD4^+^, CD8^+^ lymphocytes T as well as Th17 cells [108]. It appears that both Th1 and Th2 are involved in SSc pathogenesis, although each population dominates in a different stage of the disease: Th2 cells in early stage and Th1 cells later in the course of SSc [43]. Both Th1 and Th2 induce inflammatory reaction, but exacerbated fibrosis occurs when prevalence of Th2 cells and production of IL-4, IL-5 and IL-13 occurs (as a direct mechanism of collagen synthesis augmentation). Th1 lymphocytes attenuate collagen production and cause collagen degradation [123].

The number of Th17 cells and their most prominent product IL-17 are elevated in patients with SSc [130]. IL-17 retains pro-fibrotic state of various cells, impacts differentiation of fibroblasts, inhibits autophagy and exacerbates overall inflammatory status, implicating its role in the pathogenesis of SSc [54, 64, 114, 121]. Adipokines are strongly linked with Th17 cells differentiation. Adiponectin, which is protective in SSc, suppresses Th17 cells differentiation [133]. On the contrary, leptin and resistin-like molecule α (RELM-α) have the ability to promote pathogenic Th17 cell response [90, 100].

Macrophages can be divided into M1 and M2 subtypes. M1 macrophages stimulate inflammatory processes mostly by IL-1, IL-6, IL-12 and TNF-α, while M2 macrophages after stimulation via IL-4, IL-10, IL-13 decrease inflammation and promote tissue repair [78]. Adipokines have an impact on the proliferation of both M1 and M2 population, thus contributing in the course of the disease.

### Adipocyte–myofibroblast transition and epithelial–mesenchymal transition

Fibrous tissue in SSc is a product of myofibroblasts. However, the source of myofibroblasts in the skin of patients with SSc is still discussed. One theory suggests that myofibroblasts are derived from pericytes released from damaged vessels [99]. Another, that myofibroblasts originate from adipocytes which undergo adipocyte–myofibroblast transition (AMT) [74]. The reason of adipose tissue loss is not only apoptosis, thus AMT process may be an explanation for adipocytes depletion in SSc [122]. This thesis stays in conformity with histological images of skin lesions in SSc, in which increase of myofibroblasts amount is at the expense of adipose tissue loss [29]. There are many similarities between AMT and epithelial–mesenchymal transition (EMT), which is a process whereby cells acquire mesenchymal features, loose intercellular contact and gain ability to migrate [135]. Partial EMT-like changes may contribute to fibrosis in the skin of patients with SSc [86]. Interestingly, differentiation of fibroblasts into myofibroblasts is a reversible process, giving a potential possibility to invert it and find new drugs attenuating fibrosis [129]. It was proved that many adipokines, especially visfatin and progranuline are involved in the process of EMT, which is a transition akin to AMT process [21, 128].

### PPARs and TLRs—main receptors involved in regulation of adipokines synthesis

Peroxisome proliferator-activated receptors (PPARs) are nuclear receptors which regulate gene expression by binding to DNA structure. A pleiotropic effect of their activation involves whole body homeostasis and metabolism [60]. The family of these consists of three subtypes, PPAR-α, PPAR-β and PPAR-γ. All subtypes of PPAR receptors are expressed in adipocytes, but PPAR-γ is believed to be crucial in inducing adipocyte differentiation and synthesis of adipokines [109]. PPARs agonists increase anti-inflammatory and lower pro-inflammatory levels of adipokines; in adipocytes, ligand activation of PPAR-γ was associated with increased production of adiponectin and decreased production of resistin and leptin [65]. It was indicated that PPAR-γ stimulation in non-adipocyte cells leads to an increased concentration of adiponectin and visfatin, while synthesis of resistin is decreased [82, 93, 122]. Adipokines also affect PPARs backward, what might be considered as a feedback loop mechanism—leptin has been proved to lower expression of PPAR-γ receptors in macrophages [16]. An experimental therapy with PPARs agonist was shown to attenuate fibrosis and this fact makes PPARs a promising therapeutic target in SSc [9].

**Fig. 1:**
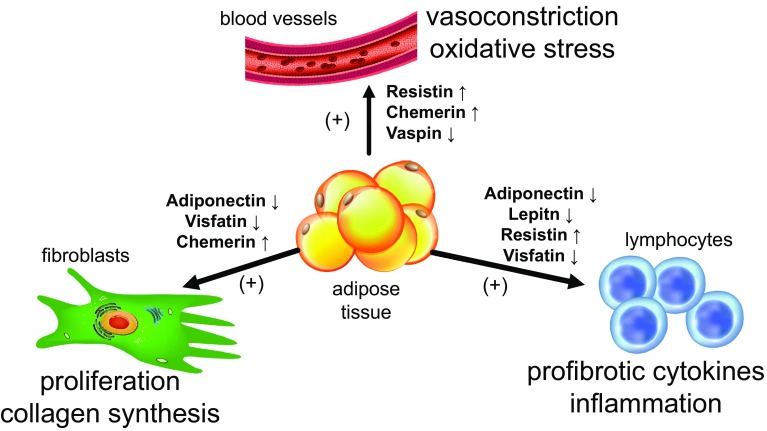
The role of adipokines in the active phase of systemic sclerosis and their effect on immune cells, fibroblasts and blood vessels. The main source of adipokines is the adipose tissue. Adipokines have a pleiotropic effect on cells involved in the pathogenesis of systemic sclerosis. Elevated serum level of resistin and chemerin as well as a decreased level of vaspin result in vasoconstriction and oxidative stress in the endothelial cells. Increased concentration of chemerin and decreased levels of adiponectin and visfatin stimulate proliferation of fibroblasts and excessive synthesis of extracellular matrix proteins. Activation of lymphocytes and the production of pro-fibrotic cytokines are augmented as a result of decreased concentration of adiponectin, leptin and visfatin and increased concentration of resistin

Toll-like receptors (TLRs) recognize both exogenous (fragments of pathogens) and endogenous (products derived from injured tissues) ligands [19]. Ten members of TLR family TLR 1–10 can be distinguished. TLR4 expression is dominating in adipose tissue, however, the presence of TLR1, TLR4, TLR7 and TLR8 was also reported. TLRs localized on adipocytes are capable of influencing the course of SSc due to stimulation of inflammation via molecular pathways activating NF-κB transcription factor and modulation of immune response [117]. TLRs agonists may induce endotheline 1 (ET-1) upregulation [27], which was reported to implicate in the vascular complications and fibrosis in SSc. TLR4 activation in particular is postulated to result in persistent fibrosis [13]. That fact may open new possibilities of targeted therapy in SSc (Fig. 1).

It was indicated that low adiponectin level in SSc patients resulted in raised macrophage M1 activity via TLR [127]. In addition, leptin upregulates TLR2 in monocytes and potentiates inflammation, while chemerin induces TLR4 in synovial fibroblasts [25, 48]. Some adipokines, like resistin, may also act as TLR agonist [12].

## Adipokines in systemic sclerosis

### Adiponectin

Adiponectin (APN) is a 30 kDa hormone. The main source of APN is adipose tissue, although other cells such as endothelium, fibroblasts, leukocytes and macrophages are capable of synthesizing APN [33]. There are three main forms of APN, depending on its post-translational modification: low-molecular-weight (LMW) trimers, medium-molecular-weight (MMW) hexamers and high-molecular-weight (HMW) multimers [118]. Globular adiponectin is another isoform, which is formed through the cleavage by macrophage and leukocyte elastase. It is significant in case of measuring APN level in serum, since this isoform may interfere with laboratory results due to elastase activity in skin lesions [119]. APN may induce IL-10 and IL-1 receptor antagonist (IL-1Ra) release in monocytes. It was shown that HMW and globular APN enhanced, while LMW reduced production of IL-6 [106]. Therefore, it is postulated that each of isoform acts pro- or anti-inflammatory, respectively. The measurement of total adiponectin level in serum is widely described in the literature. However, the level of each isoform may differ and might be also taken into consideration in the terms of the development of the disease, including SSc.

Metabolic syndrome and related diseases are associated with HMW, while LMW is more closely linked with autoimmune phenomena [66]. Interestingly, TNF-α impairs adiponectin multimerization and decreases adiponectin secretion, which may explain different ratios between APN isoforms in these disorders [40].

Two receptors for adiponectin have been distinguished, ADIPOR1 and ADIPOR2. APN polarizes macrophages into M2 subtype by ADIPOR2 stimulation which results in an increased level of pro-fibrotic interleukines [73, 87]. However, APN has also a role in polarization of CD4+ lymphocytes into Th1 and preventing from Th2 subtype dominance [49]. A decreased APN level was observed mostly in the early stage of SSc, resulting in a prevalence of Th2 lymphocytes and their pro-fibrotic action in this stage. In the later phase, an increased level of APN was found [59], suggesting Th1 cells and their anti-fibrotic effect domination. Apart from Th polarization mechanism, APN seems to have a direct impact on fibroblasts and fibrosis. Fibroblast-binding APNs demonstrate anti-fibrotic effect via activation kinase involved in inflammation (AMPK) [26]. In human skin biopsy, cellular phosphorylated AMPK level which reflects APN activity in fibrotic tissue was considerably decreased in SSc patients compared to healthy control [75]. Studies showed that APN also inhibited EMT in breast and prostate cancer, which indicates a restrain of EMT process in SSc due to increased APN level [47, 111]. Moreover, an inverse correlation between adiponectin level and the modified Rodnan skin score (mRSS) was found [8, 59]. Adiponectin, as an anti-fibrotic molecule, may be positively correlated with disease duration and its decreased level seems to be one of the factors exacerbating fibrosis in the early stage of SSc [59].

### Leptin

Leptin belongs to the type I cytokine superfamily and its structure is similar to IL-2, IL-6 and G-CSF [132]. First reports about leptin focused on its role in overweight and as a potential goal for obesity therapy [134]. In the hypothalamus, leptin suppresses appetite and plays a major role in sustaining body weight as well as energy homeostasis [103]. Serum leptin level is associated with the amount of adipose tissue in the human body [102]. Adipocytes are not the only source of leptin; it is also produced in large amounts by fibroblasts, keratinocytes and endothelial cells [96]. High-fat diet, estrogens and pro-inflammatory cytokines enhance leptin production, while catecholamines, androgens and PPAR-γ agonists decrease its level [38, 51, 102]. Apart from the role of leptin in obesity, it is involved in developing other disorders such as type 1 diabetes, systemic lupus erythematosus and psoriasis. This may be due to affecting angiogenesis, extracellular matrix (ECM) production and enhancing inflammation [88]. Leptin acts by ObR—leptin receptor. Measurement of serum leptin receptor level may be useful in clinical practice; it was indicated that frequency of esophageal reflux was significantly lower in SSc patients with elevated than in those with reduced level of serum leptin receptor (6.3% vs. 35.3%, respectively) [88].

Moreover, an elevated serum level of leptin receptor could be a clinically useful marker of systemic sclerosis spectrum diseases (SSDs) and its measurement over time in patients with SSD may lead to early detection of SSc [88]. Although ObR is different from APN receptor, they act through the common signaling pathway. Despite similarity of signaling pathways, leptin may antagonize the activity of APN. Leptin has been associated with either acute or chronic phase of inflammation. As a cytokine, leptin affects the immune system in several ways. In murine model, leptin increased production of antinuclear antibodies [70]. Moreover, leptin polarizes Th lymphocytes into Th1 and suppress Th2 phenotype, thus enhances production of pro-inflammatory cytokines [77]. In keratinocytes, leptin increases production of IL-6, IL-8 and TNF-α as well as expression of genes related to wound healing such as metalloproteinase-1 (MMP-1) [61, 126]. Furthermore, leptin enhances subtype M1 macrophage growth and differentiation [2]. Leptin activates endothelial cells and acts as a chemokine, causing attraction of macrophages into adipose tissue, hence it is involved in creating a local inflammatory niche in patients with SSc [20, 24]. The research indicated that leptin levels are significantly lower in patients with the active phase of SSc than in inactive (1.92 ± 2.90 vs. 7.02 ± 7.65 ng/ml) and this fact harmonizes with the theory of Th2 lymphocytes domination in the early, active stage with enhanced fibrosis and Th1 in the latter inactive stage of SSc [15]. On the contrary, it was also shown that circulating leptin level does not significantly differ between patients with SSc and healthy controls [63]. Taking into consideration each adipokine individually may be questionable. In other diseases associated with extended fibrous tissue production, such as idiopathic pulmonary fibrosis, it was reported that measuring adiponectin/leptin ratio is useful to assess the course of the disease and helps to predict the intensity of fibrosis [72]. Except for fibrosis, low adiponectin/leptin ratio may also indicate adipose tissue dysfunction, increased oxidative stress and inflammation [32].

Apart from inducing inflammation, leptin was associated with indirect stimulation of fibrosis. Binding leptin to ObR in regulatory T cells (Tregs) is a signal for hyporesponsiveness and decreased proliferation of Tregs which was indicated to enhance fibrosis [22]. In addition, leptin induces human lung fibroblast to transdifferentiate into myofibroblasts responsible for collagen production [17]. Leptin promotes pulmonary fibrosis also by inhibiting autophagy via PI3K/Akt/mTOR pathway. Interestingly, mTOR inhibitors reversed this effect, therefore rapamycin could be considered a therapeutic agent in pulmonary fibrosis in SSc [36]. Leptin may promote fibrosis in an alternative mechanism, which involves renin–angiotensin system (RAS). In the central nervous system, leptin stimulates hypothalamic melanocortin system, increases sympathetic activity and leads to RAS activation [125]. What is more, in the elderly, leptin was associated with arterial stiffness [35], which is one of the features of vascular involvement in SSc [113]. Therefore, leptin could be used as a potential marker of vascular damage in SSc, however further investigations are required (Table 1).

**Table 1:** Potential role of adipokines in systemic sclerosis

Adipokine	Level in SSc comparing to controls	Source of sample	Correlation with complications
Adiponectin	Decreased—only in the early stage of SSc [59]	Serum level, mRNA and skin biopsy	Negative correlation with Valentini activity score [89] and mRSS [59]
Leptin	Decreased (active vs. inactive SSc) [15]	Serum level	↑ Pulmonary fibrosis [36] and pulmonary arterial hypertension [46] ↑ Arterial stiffness [35]
	Increased (dominating) [95]	Serum level	
Resistin	Increased (no statistical significance) [95]	Serum level	↑ Pulmonary arterial hypertension [79] Digital ulcers biomarker [79] Positive correlation with digital vasculopathy [89] Chronic kidney disease biomarker [10]
Visfatin	Increased in late stage dcSSc [80]	Serum level	Positive correlation with regression of skin lesions [80]
Chemerin	Increased [6]	Lesional psoriatic vs. uninvolved skin	Positive correlation with disease duration [5] Positive correlation with mRSS [5]
	Increased [5]	Dermal blood vessels	
	Decreased [5]	Dermal fibroblasts	
Vaspin	Decreased [92]	Serum level	Biomarker of digital ulcers in SSc [84]
Adipsin	Increased [57] (significantly higher in lcSSc vs. dcSSc)	Serum level	Positive correlation with frequency of anti-centromere antibodies [57] Negative correlation with frequency of anti-Scl-70 antibodies [57] Positive correlation with SSc-PAH [57]
Apelin	Decreased [131]	Fibroblasts	Negative correlation with mRSS [131] Biomarker of digital ulcers [7, 131] Biomarker of acro-osteolysis [131] Positive correlation with prevalence of scleroderma renal crisis and PAH in late stage SSc [7]
	No difference [7, 131]/increased in early stage of SSc vs. later stages [7]	Serum level	
Omentin	No difference [83]	Serum level	Positive correlation with disease duration in dcSSc [83] Positive correlation with right ventricular systolic pressure [83] (possible biomarker of SSc-PAH/vascular involvement)
	Decreased in dcSSc vs. lcSSc [83]	Serum level	
CTRP-3	Not studied in SSc yet	–	–

### Resistin

Resistin belongs to the found in inflammatory zone (FIZZ) family of proteins which means that it is overexpressed in the area of inflammation. In contrast to other adipokines, it is mainly secreted by monocytes in humans, not adipocytes [62]. Resistin is involved in human lipid, glucose and insulin homeostasis. A correlation between resistin level and obesity together with metabolic syndrome has been found. Resistin seems to be a molecular biomarker of inflammation and disorders related to metabolic syndrome such as cardiovascular disease or diabetes [1]. Due to the fact that resistin is primarily secreted by macrophages, it may have a direct impact on inflammation. Despite having a significant contribution in the maintenance of energy homeostasis, resistin may exert a larger impact on inflammatory processes—resistin has been linked to rheumatoid arthritis, psoriatic arthritis and systemic lupus erythematosus and is suspected to be involved in SSc pathogenesis [89]. It is widely known that inflammation triggers fibrosis. Hence, resistin was indicated to exacerbate fibrosis. However, the meta-analysis of four studies considering resistin levels in SSc does not exhibit its significantly different level compared to healthy individuals [63].

Mechanism of pro-inflammatory action of resistin involves TLR4. Intracellular signals are mediated via NF-κB and MAPK and lead to IL-1, IL-6 and IL-8 secretion [112]. Furthermore, these pro-inflammatory cytokines increase the expression of resistin in human mononuclear cells [14]. This triggers a positive feedback loop of self-damaging process, where resistin stimulates pro-inflammatory cytokines’ secretion and pro-inflammatory cytokines elevate resistin’s concentration. Resistin crosses brain–blood barrier and binds to TLR4 receptors in hypothalamus resulting in systemic low-grade inflammation and insulin resistance [12]. However, the role of TLR as a link between SSc and adipokines has not been clearly established yet and requires further research.

It was proved that not only TLR4 but also PPAR-γ is associated with the concentration of resistin in the serum. Studies showed that resistin level may be decreased by TLR4 antibodies or PPAR-γ agonists [93, 112] providing another argument, that these molecules could be considered as potential therapeutic agents for SSc treatment.

It is worth highlighting that resistin modulates vessels activity. Vasoactive mechanism of resistin is mediated through enhancing production of ET-1 [116]. This results in vasoconstriction along with inflammatory cells attraction and diapedesis through a vessel wall. Smooth muscle cells proliferation, endothelial cells migration as well as adjacent tissue remodeling via metalloproteinases induced by resisitn [28] may additionally potentiate vasoconstriction present in patients with SSc [79]. Development of pulmonary arterial hypertension (PAH) was linked to role of resistin-induced angiogenesis and immune response [79]. Furthermore, it was indicated that prevalence of digital ulcers tended to be higher in SSc patients with elevated serum resistin levels than in those with non-elevated levels (50% vs. 16%, respectively) [79]. Resistin stimulates P-selectin expression on human platelets, facilitating thrombus formation [98]. Moreover, the level of resistin was positively correlated with plasminogen activator inhibitor 1 (PAI-1), which is a pro-fibrotic molecule [97]. This may be one of the factors explaining higher prevalence of deep vein thrombosis and pulmonary thromboembolism in SSc patients than in general population (10.5- and 7.00-fold higher than in control group, respectively) [18].

### Visfatin

Visfatin (pre-B-cell colony-enhancing factor, PBEF) is predominantly secreted by visceral adipose tissue. In human, it is correlated with metabolic disorders along with obesity. This adipokine is a PPAR-γ depended protein and activating this molecular pathway results in inducing visfatin gene expression in macrophages, mostly M1 subtype localized in adipose tissue, but not in adipocytes [82, 124]. Effect on the immune system is confirmed by the fact that lack of visfatin expression strongly attenuated the development of both T- and B-cell lymphocytes [110]. Visfatin acts as a pro- and anti-inflammatory cytokine; it was indicated to up-regulate circulating TNF-α, IL-1β, IL-6 as well as IL-4, IL-10 and IL-1Ra [124]. Visfatin is also capable of increasing levels of cell adhesion molecules ICAM-1, VCAM-1 and E-selectin in endothelial cells [110]. Interestingly, pro-inflammatory activity of visfatin was abolished when insulin receptor signaling was blocked [82]. Concentration of visfatin in the serum is comparable among total SSc patients, diffuse cutaneous SSc (dcSSc), limited cutaneous SSc (lcSSc) and healthy individuals. It has been found that an increase of visfatin level in serum is accompanied by regression of skin lesions in late-stage dcSSc (> 6 years duration) [80]. The proposed mechanism of this phenomenon is both visfatin-induced Th1 polarisation and the direct anti-fibrotic effect on dermal fibroblast [80].

### Chemerin

Chemerin is an immunomodulating protein secreted predominately by adipose tissue as well keratinocytes in basal and suprabasal layers of the skin [81]. Chemerin was associated with inflammatory processes in systemic sclerosis, rheumatoid arthritis, systemic lupus erythematosus and psoriasis. Moreover, an inverse correlation between chemerin and body mass index was found [37]. This finding suggests that production of chemerin by non-adipose tissue is independent from the amount of the body fat. Chemerin receptor (ChemR23) is localized on the surface of endothelial cells, and its expression is regulated by TNF-α, IL-1β and IL-6. Signaling pathways involved in transducing signal via ChemR23 promote endothelial cell proliferation and migration, leading to endothelial barrier dysfunction [76]. Serum proteases have the ability to cleave chemerin resulting in the formation of different isoforms, which bind to macrophages and stimulate secretion of pro-inflammatory cytokines (TNF-α, IL-6 and IFN-γ). Moreover, these isoforms of chemerin seem to recruit plasmacytoid dendritic cells (pDCs) and NK cells. PDCs combat pathogens, although they may exacerbate inflammation in skin lesions and were associated with fibrosis in SSc patients [3]. Furthermore, it was shown that in other cutaneous diseases such as psoriasis, mRNA levels of chemerin were elevated in lesional skin compared to uninvolved skin of the same patients. Probable mechanism involves accumulation of chemerin which results in potentializing attraction of inflammatory cells [6].

The pro-inflammatory nature of chemerin is confirmed by the fact that adalimumab (TNF-α inhibitor) therapy was indicated to lower chemerin level, which was linked with reduction of rheumatoid arthritis disease activity and levels of pro-inflammatory cytokines [31].

It was indicated that serum chemerin level in patients with SSc is elevated compared to healthy individuals. However, renal dysfunction has an influence on serum concentration in both SSc patients and control group. Chemerin seems to be involved in the development of the skin sclerosis in the early stage of SSc (disease duration < 1 year). Moreover, chemerin level was positively correlated with mRSS [5]. Association between serum chemerin level and presence of digital ulcers in SSc patients was also reported to date and may be a proof of increased chemerin expression in dermal blood vessels [5].

It is important to mention that increased chemerin level was associated with risk of impaired renal function (OR 2.72). It can be explained by direct damage of kidneys or reduced chemerin clearance in patients with SSc but also in a general population [136]. Thus, this can imply the emerging role of chemerin in internal organ disorders in SSc.

### Vaspin (visceral adipose tissue-derived serine protease inhibitor)

Vaspin is an adipokine expressed predominantly in visceral adipose tissue and plays important roles in metabolic syndrome and its relevant vascular complications. Serum vaspin levels were lowered in sera of patients with SSc, however, no statistical significance was observed (341 ± 284 vs. 434 ± 343 pg/mL, *p* > 0.05) [92]. Despite no difference between SSc and control group, serum vaspin levels were significantly decreased in SSc patients with digital ulcers compared with those without, suggesting a protective role of vaspin to digital ulcers development [84]. Therefore, vaspin may have protective properties against endothelium dysfunction in SSc and its level may reflect endothelium condition in SSc patients. No correlation of serum vaspin levels with C-reactive protein and erythrocyte sedimentation rate was found, which did not indicate a significant role of vaspin in the inflammatory process in SSc [84]. It can be explained by the fact that vaspin, interacting with NF-κB pathway was able to inhibit pro-inflammatory cytokines signaling such as TNF-α, IL-1, IL-6 [69]. Moreover, vaspin suppresses the expression of other adipokines: leptin and resistin [41].

### Novel adipokines

Adipsin (complement factor D) is a serine protease secreted by adipocytes. It has the ability to activate the alternative pathway of complement. Increased complement activity [107] and, as a result, the membrane attack complex on arterioles were observed in SSc patients [105]. Early administration of eculizumab, which is terminal complement inhibitor, manifested therapeutic potential in patients with renal crisis in the course of SSc [23]. Elevated serum level of adipsin in SSc was associated with vascular involvement, in particular PAH and is considered as a potential biomarker in this complication related to SSc [57].

Apelin is an endogenous ligand of G protein-coupled receptor called APJ. It is widely expressed in central nervous system and many peripheral tissues, including adipose tissue [131]. Recent studies reported that Apelin/APJ complex alleviates renal, myocardial and lung fibrosis [45]. It was also showed that skin fibrosis induced by TGF-β is inhibited by apelin, but precise mechanisms of this action requires further investigation [131]. Expression of apelin was significantly reduced in SSc fibroblasts compared to normal fibroblasts and serum apelin levels were negatively correlated with mRSS. However, the differences of serum apelin levels between SSc patients and control group vary among studies [131], but there was no statistically significant difference of serum apelin levels between SSc patients and control group [7, 131]. In the context of the still unclear role of apelin, further researches are required to establish its position in SSc pathogenesis.

Omentin is a newly identified adipokine that is produced abundantly by visceral adipose tissue. It was reported that omentin has anti-inflammatory properties acting via AMPK signaling pathway and has a potent vasodilatory effect by activating endothelial nitric oxide synthase (eNOS). Omentin suppresses inflammatory responses in endothelial cells, inhibits chemoattraction and production of ECM [120]. So far, only one study has investigated omentin and its role in SSc pathogenesis and clinical outcome [83]. Serum concentration of omentin was comparable between SSc patients and healthy control. However, omentin level was decreased in dcSSc patients compared to lcSSc patients (715.4 vs. 980.2 ng/mL, *p* = 0.013). What is more, omentin level in dcSSc patients was positively correlated with disease duration (≤ 5 vs. > 5 years, *p* = 0.035). Increased right ventricular systolic pressure was observed in SSc patients with elevated omentin concentration indicating a possible role of omentin as a biomarker of vascular involvement leading to SSc-related PAH (SSc-PAH) [83].

CTRP-3 (C1q/TNF-related protein) is one of emerging adipokines, whose molecular structure and function resemble adiponectin. It was indicated that CTRP-3 exerts anti-inflammatory and anti-fibrotic effects in fibroblasts. Reduced fibrotic activity caused by CTRP-3 is a consequence of targeting connective tissue growth factor (CTGF) and collagen I expression as well as TGF-β production [44]. In rat model, CTRP-3 ameliorated TGF-β-induced expression of CTGF, proliferation, migration and collagen synthesis of adventitial fibroblasts [67]. CTRP-3 demonstrates a beneficial effect on the cardiovascular system potentially ameliorating pathological vascular remodeling [104]. Although there were only a few reports indicating the role of CTRP-3 in fibrosis, current studies suggest CTRP-3, similarly to adiponectin [94] and adipsin [57], may tie together adipokines, fibrosis and complement system. The exact position of CTRP-3 still awaits to be discovered, as there is a lack of research concerning its role in SSc pathogenesis (Table 2).

**Table 2:** Potential mechanism of adipokines’ action in SSc pathogenesis and their role in inflammation

Adipokine	(Potential) Mechanism of action	Role in inflammation
Adiponectin	HMW and globular APN : ↑ TNF-α, IL-8, IL-6 [39, 106] LMW: ↓ IL-6, ↑ IL-10 [85] M2 macrophage polarization [87] ↑ Th1/Th2 ratio [49] ↓ Differentiation of Th17 [133]	Anti-inflammatory (dominating) [85, 133]
Leptin	↑ IL-6, IL-8, TNF-α [126] ↑ MMP-1 [61] ↑ Th1/Th2 ratio [77] ↑ Th17 cell response [100] ↓ Treg proliferation and activity [22] Chemoattractant [20] ↓ Autophagy [36] RAS activation [125]	Pro-inflammatory [20, 22, 100, 126]
Resistin	↑ IL-6, IL-8 secretion [112] ↑ ECM production (via CTGF) [50] ↑ Th17 cell response [90] ↑ ET-1 production [116] → vasoconstriction ↑ Blood clot formation [98]	Pro-inflammatory [90, 112, 116]
Visfatin	↑ IL-4, IL-10, IL-1Ra circulating levels [124]	Anti-inflammatory (dominating) [124]
	↑ Development T- and B-cell lymphocyte [110] ↑ Th1/Th2 ratio [91] ↑ TNF-α, IL-1β, IL-6 [124] ↑ ICAM-1, VCAM-1, E-selectin, IL-6, IL-8 in endothelial cells [110]	Pro-inflammatory [110, 124] – abolished by blocking insulin receptor signaling [82]
Chemerin	M1 macrophage polarization [68] ↑ TNF-α, IL-6, IFN-γ [68] ↑ PDCs, macrophages, NK cells attraction [6, 11] ↑ IL-10 [68]	Pro-inflammatory [6, 11, 68] Anti-inflammatory [68]
Vaspin	↓ TNF-α, IL-1, IL-6, ICAM-1, VCAM-1, MCP-1 [69] ↓ Leptin and resistin [42]	Anti-inflammatory [42, 69]
Adipsin	Mediated via complement pathway activation [57]	Pro-inflammatory [57]
Apelin	↓ TGF-β-induced skin fibrosis [131] ↑ VCAM-1 and MCP-1 [71] ↑ Angiogenesis [71] ↓ TNF-α, IL-1, MCP-1, MIP-1α, IL-6 [71]	Pro-inflammatory [71] Anti-inflammatory [71] Pro- and anti-fibrotic in context-dependent manner [7]
Omentin	M2 macrophage polarization [120] ↓ ICAM-1, VCAM-1, TNF-α, IL-6, MCP-1, COX-2 [120] ↑ eNOS [120] – vasodilatation ↓ Chemoattraction [120] ↓ ECM production [120]	Anti-inflammatory [120]
CTRP-3	↓ TLRs pro-inflammatory effects [44] ↓ CTGF, TGF-β, collagen synthesis [44, 67] ↓ Proliferation, migration of fibroblasts [67]	Anti-inflammatory [44]

## Conclusions

Adipose tissue is associated with SSc both in anatomic and functional manner. Although adipokines have been profoundly studied in many disorders, such as diabetes, metabolic syndrome and psoriasis, further investigations are required to establish their link with SSc. Adipokines may be linked with the course of SSc and its possible complications. Moreover, adipokines seem to influence the TLRs and PPARs signaling and expression. This suggests a pivotal role of both receptors in SSc pathogenesis. This review gives an insight into the complex role of adipokines in SSs which are involved in many processes such as immunomodulation and fibrosis. Adipokines are a heterogenic group of molecules; leptin, resistin and chemerin present a potential for triggering inflammation, while adiponectin, visfatin and vaspin exhibit mostly contradictory anti-inflammatory features. Novel adipokines such as adipsin, apelin, omentin and CTRP-3 appear also as molecules involved in SSc pathogenesis. In the future, adipokines may be useful as predictive and diagnostic factors in SSc. However, further researches are vital to establish the firm correlation between adipokines and SSc.
